# Effect of vibration associated with cryotherapy on vaccine-related pain and anxiety levels in adults: study protocol for a randomized clinical trial

**DOI:** 10.1186/s13063-022-06564-7

**Published:** 2022-08-01

**Authors:** Elaine Aparecida da Cunha Lima, Luana Vieira Toledo, Marisa Dibbern Lopes Correia, Daniela de Almeida Pereira, Renata Oliveira Caetano, Thaís Bitencourt Faria, Luciene Muniz Braga

**Affiliations:** 1 Department of Medicine and Nursing, Universidade Federal de Viçosa, Viçosa, Minas Gerais Brazil; 2 Health Division, Federal University of Viçosa, Viçosa, Minas Gerais Brazil

**Keywords:** Vaccination, Pain, Anxiety, Pain management, Cryotherapy, Nursing

## Abstract

**Background:**

Vaccination is one of the most effective strategies for prevention and eradication of immunopreventable diseases, but community acceptance of vaccination can be influenced by different factors, such as pain and anxiety. The use of high-frequency vibration associated with cryotherapy has been used to manage pain and anxiety during the vaccination process in children, but studies with adults are still scarce. This study aims to evaluate the effect of high-frequency vibration associated with cryotherapy on the levels of self-reported pain and anxiety related to administration of the Influenza vaccine intramuscularly in adults.

**Methods:**

A two-arm, parallel, randomized clinical trial conducted in a Brazilian Primary Health Care Unit is proposed. A sample of 350 adults will be randomly assigned to participate in the control group, receiving the vaccine intramuscularly according to the standard protocol of the service, or in the intervention group, receiving the vaccine by the same route and using a portable device of high frequency vibration associated with cryotherapy for 30 s before and during administration. The primary endpoints will be self-reported levels of pain, assessed before and after vaccine administration. Secondary endpoints will be levels of anxiety, satisfaction with vaccine administration, and discomfort caused by high frequency vibration and temperature of the frozen bag in contact with the skin. Self-reported levels of pain and anxiety will be compared before and after vaccination as well as between the control and intervention groups.

**Discussion:**

By evaluating the effect of high-frequency vibration associated with cryotherapy on pain and anxiety levels, we expect to find evidence that will support nursing practice, in order to promote greater comfort and safety in the vaccination process and, consequently, greater compliance by the population, by minimizing its undesirable effects.

**Trial registration:**

Human Research Ethics Committee Opinion Number: 5.138.564. Approved on December 2, 2021. Brazilian Registry of Clinical Trials (REBEC): Registration number RBR-5zgy25w. Registered on December 09, 2021.

**Supplementary Information:**

The online version contains supplementary material available at 10.1186/s13063-022-06564-7.

## Administrative information

Note: the numbers in curly brackets in this protocol refer to SPIRIT checklist item numbers. The order of the items has been modified to group similar items (see http://www.equator-network.org/reporting-guidelines/spirit-2013-statement-defining-standard-protocol-items-for-clinical-trials/).

**Table:** 

Title {1}	Effect of vibration associated with cryotherapy on vaccine-related pain and anxiety levels in adults: study protocol for a randomized clinical trial
Trial registration {2a and 2b}.	The Brazilian Clinical Trials Registry (ReBec): RBR-5zgy25w
Protocol version {3}	03/17/2022 Version 1.0
Funding {4}	The study was financed with own resources.
Author details {5a}	Elaine Aparecida da Cunha Lima: elaine.aparecida@ufv.br^1^ Luana Vieira Toledo: luana.toledo@ufv.br^1^ Marisa Dibbern Lopes Correia: marisa.lopes@ufv.br^1^ Daniela de Almeida Pereira: danalmeidap@yahoo.com.br^2^ Renata Oliveira Caetano: renata.o.caetano@ufv.br^1^ Thaís Bitencourt Faria: thais.bitencourt@ufv.br^1^ Luciene Muniz Braga: luciene.muniz@ufv.br; ^1^ ^1^Department of Medicine and Nursing, Federal University of Viçosa, Minas Gerais, Brazil ^2^Health Division, Federal University of Viçosa, Minas Gerais, Brazil.
Name and contact information for the trial sponsor {5b}	The study was financed with own resources. There is no sponsor.
Role of sponsor {5c}	No sponsor of the study, the resources were two own researchers.

## Introduction

### Background and rationale {6a}

Influenza is an acute viral infection that affects the respiratory system [1, 2]. The severity and impact of the disease are associated with the virulence and the degree of transmissibility of the virus, as well as the adherence to preventive measures by the community [3].

It is estimated that each year, 20% of the world’s population is infected, with severe Influenza infection being one of the leading causes of morbidity and mortality worldwide [4]. The largest pandemic historically recorded occurred in 1918 and was caused by the influenza A H1N1 virus, known as the Spanish flu, responsible for more than 20 million deaths worldwide [5]. In 2009, again the influenza A virus caused the first pandemic of the twenty-first century, with mortality of almost 400,000 individuals [3].

Among the measures of prevention and control, the annual vaccination against influenza virus, adopted by several countries [3, 6], stands out. In Brazil, the National Immunization Program annually coordinates the national vaccination campaign since 1999 [7]. However, despite the importance of vaccination, it is found that in different countries, the campaign against Influenza has low adherence. In 2017, in Spain, only 40.3% of the population with chronic conditions received the immunization [8]. Similarly, in 2021, in China, only 4.7% of the elderly aged 60 years and older were vaccinated [9]. In the national context, vaccination coverage has been advancing from 70% in 1999 to 82.8% in 2020 [10]. However, despite the positive increase in the vaccinated population, 50,482 cases of Influenza infection were confirmed in 2009 [5] and 2.150 in 2020 [10].

The causes for people’s non-vaccination or hesitation are complex and diverse issues, as they involve social, cultural, and economic aspects and may vary from one place to another [11]. Among the main causes are misinformation (73.9%) [12, 13], fear of the needle, and adverse events (55.4%) [14].

The fear of the needle is a result of the pain generated during application in the muscle, being considered a personal and subjective experience. This individual perception can be influenced, in variable degrees, by biological, psychological, and social factors, with consequent anxiety in future immunizations [15].

Vaccine-related pain and anxiety have gained prominence on the international scene. In 2015, the World Health Organization (WHO) issued a position paper on this issue and presented a variety of feasible evidence-based, mostly non-pharmacological, accessible, easy to perform, low or no cost, training- and time-intensive interventions, such as tactile stimulation, distraction, high-frequency vibration, and cryotherapy (ice pack) [16].

The use of high-frequency vibration associated with cryotherapy as a non-pharmacological strategy for the relief of pain related to needle procedures has been used, especially in the pediatric population [17–20]. A randomized clinical trial (RCT) demonstrated that during vaccination, the group of children who received high-frequency vibration associated with cryotherapy had lower mean reported pain scores than children who did not receive this intervention (3.56 vs 5.92—*p* = 015,0) [21]. In adults, two international studies demonstrated the effectiveness of the portable high-frequency vibration device associated with cryotherapy on pain and anxiety levels during intramuscular (IM) vaccine administration [22]. However, there is a lack of national studies aimed at evaluating the effect of this intervention in the general adult population.

Thus, controlled clinical trials of high methodological quality are necessary to evaluate the effect of non-pharmacological nursing interventions such as high-frequency vibration associated with cryotherapy on the levels of self-reported pain and anxiety related to the administration of influenza vaccine by IM in adults. The results of this study may support the reorientation of nursing practices in order to promote greater comfort for the population and safety in the vaccination process. Thus, it is expected to improve the adherence of adults to immunization campaigns by minimizing undesirable effects during administration, especially pain.

### Objectives {7}

The general objective of this study is to evaluate the effect of high frequency vibration associated with cryotherapy on the levels of self-reported pain and anxiety related to the administration of the Influenza vaccine by IM in adults. In addition, it has as secondary objectives:

1. To evaluate the level of self-reported pain expected by adults before administration of the Influenza vaccine by IM with and without the use of high-frequency vibration associated with cryotherapy
2. To evaluate the level of pain self-reported by adults after administration of the Influenza vaccine by IM with and without the use of high-frequency vibration associated with cryotherapy
3. To evaluate the level of anxiety self-reported by adults before and after administration of the Influenza vaccine by IM with and without the use of high-frequency vibration associated with cryotherapy

### Trial design {8}

This is a parallel, open, two-armed RCT, with allocation of participants in a 1:1 ratio and structured to evaluate the superiority of the use of a portable high-frequency vibration device associated with cryotherapy, on the levels of pain and anxiety related to the administration of the influenza vaccine (intervention group—IG), compared to the absence of its use (control group—CG).

## Methods: participants, interventions, and outcomes

### Study setting {9}

The study will be conducted in a vaccination room of a Basic Health Unit located on the premises of a public Brazilian higher education institution. This is a service that offers multidisciplinary outpatient care with doctors, nutritionists, nurses, dentists and speech therapists, directed to students, teachers, and technical-administrative servers of that institution.

The vaccination room is accredited with the Brasilian Ministry of Health and open to the entire community. The team of professionals working in vaccination consists of a nurse, a nursing technician and a nursing assistant. The national immunization program provides nineteen vaccines for different age groups, among them the influenza, in addition to special immunobiologicals, sent to patients with contraindications for the vaccines usually administered or patients in special conditions of morbidity.

### Eligibility criteria {10}

The study population will consist of adults who come to the basic health unit to receive the influenza vaccine by the IM route during the National Immunization Campaign of the year 2022.

Participants will be included if they are 18 years of age or older, have good cognition, are literate, and exclusively receive influenza vaccine via IM in the deltoid region during the study.

Participants with previous reports of pain in the upper limbs, presence of lesions or abrasions in the deltoid region, loss or alteration of sensation in the upper limbs, and previous use of analgesics in the last six hours prior to immunization will be excluded.

### Who will take informed consent? {26a}

All potential participants will be invited to participate in the study by personal contact made by members of the study team while waiting to receive the vaccine. The researchers will explain the details of the study to the potential participants, and if they are interested in participating, they will be asked to read and sign the informed consent form.

### Additional consent provisions for collection and use of participant data and biological specimens {26b}

Not applicable. The study does not include biological data collection.

## Interventions

### Explanation for the choice of comparators {6b}

The control group (CG) will be composed of participants who received the vaccine intramuscularly, according to the standard protocol of the service, without the use of any intervention for pain relief and anxiety. This group will be compared with the intervention group (IG).

### Intervention description {11a}

Patients will be randomized and assigned to one of two groups: control group (CG) or intervention group (IG). They will remain in these groups until the end of the study.

- CG: participants who will receive the influenza vaccine by IM as per the standard protocol of the service, without the use of any intervention for pain relief and anxiety
- IG: participants who will receive the influenza vaccine by IM route using the portable high-frequency vibration device associated with cryotherapy for 30 s before application and during administration of the vaccine. The reusable plastic handheld device called Buzzy® has a reusable ice pack (cryotherapy) and a button to trigger the high-frequency vibration. The device is based on the pain gate control theory by using the non-painful stimuli of cold and high frequency vibration on the deltoid before and during vaccine administration to inhibit the transmission of painful stimuli [19]

The research will take place during the 2022 National Influenza Immunization Campaign, starting in April. The principal investigator will be responsible for administering the influenza vaccine to all participants in both groups.

The influenza vaccine consists of inactivated, fractionated, and purified viruses, characteristics that ensure that it does not cause the disease. The immunobiological distributed free of charge by the Ministry of Health will be used. The influenza vaccine (fragmented, inactivated) is an injectable suspension composed of different strains of the inactivated, fragmented, and purified Myxovirus influenzae virus, A/Victoria/2570/2019 (H1N1)pdm09; A/Darwin/9/2021 (H3N2); B/Austria/02/1359417/2021 (linhagem B/Victoria).

In both groups (control and intervention), the participants will be seated, in an air-conditioned environment, and will receive guidance about the procedure and possible adverse events. If there is no contraindication, the participants can choose which limb they want to receive the vaccine in (right or left deltoid). The researcher will perform hand hygiene and help with the correct positioning, keeping the forearm of the selected limb over the abdomen, with the aim of relaxing the shoulder.

CG: The researcher will check the integrity of the immunization liquid and expiration date of the vial with the participant. The researcher will clean the rubber of the vial with dry cotton and will attach a 25 × 7.0 dec/mm needle to the 1 ml sterile syringe and insert it into the vial. He will homogenize the liquid and then aspirate 0.5 ml of immunizer, as directed by the Health Ministry. After aspiration of the immunizing liquid, the needle will be replaced by the 25 mm × 0.6 dec/mm needle. The 25mm × 0.6dec/mm needle, attached to the syringe, will be inserted completely into the deltoid muscle at a 90° angle to the skin of the arm, then the vaccine will be administered quickly to reduce the time the needle stays in the muscle. The needle will be removed at a 90° angle, while the researcher with a cotton swab, lightly presses the muscle. After complete removal of the needle, compression will be maintained on the application site, without massage, for approximately 30 s. After this time, the presence of local reactions, such as bleeding, will be evaluated. If present, compression will be maintained until the bleeding stops completely and an antiseptic bandage will be applied.

IG: The researcher will check the integrity of the immunizer liquid and expiration date of the vial with the participant. The researcher will clean the rubber of the vial with dry cotton and will attach a 25 × 7.0 dec/mm needle to the 1 ml sterile syringe and insert it into the vial. He will homogenize the liquid and then aspirate 0.5 ml of immunizer, as directed by the Health Ministry. After aspiration of the immunizing liquid, the needle will be replaced by a 2 5mm × 0.6 dec/mm needle. An assistant researcher will remove the ice pack (cryotherapy) from the freezer at the moment of vaccine administration and will attach it to the portable vibration device. The button to trigger the vibration will be turned on, the device will be attached to the site where the vaccine will be administered (deltoid muscle) and will remain for 30 s. The device’s dwell time will be timed, using a Vollo® digital stopwatch with alarm. After this time, the attached device will be slid about 2 cm above the administration site and the vaccine will be administered by the principal investigator. The 25 mm × 0.6dec/mm needle, attached to the syringe, will be inserted fully into the deltoid muscle at a 90° angle to the skin of the arm, and then the vaccine will be administered quickly to reduce the time the needle remains in the muscle. The needle will be removed at a 90° angle while the researcher lightly presses the muscle with a cotton swab. After complete removal of the needle, compression will be maintained at the application site, without massage, for approximately 30 s. The handheld device will be turned off and removed from the limb. Local reactions such as bleeding will be evaluated. If present, compression will be maintained until the bleeding stops completely, and an antiseptic bandage will be applied. The assistant researcher will disinfect the portable high-frequency vibration device and the ice pack with 70% alcohol. The ice pack will be returned to the freezer for later use.

After vaccine administration, the syringe and needle packaging will be discarded in regular trash containers and the vaccine bottle in an appropriate place, according to legislation. The researcher will perform hand hygiene and sign the full name legibly on the vaccine card and return it to the participant, who will be instructed to remain on the premises for 15 minutes to observe for possible adverse events.

### Criteria for discontinuing or modifying allocated interventions {11b}

Vaccination is a one-time intervention, and assessment of outcomes will occur before and after administration. However, during its realization, the volunteer may be discontinued if he/she withdraws from the study. For the participants of IG, the discontinuation may occur by any change or discomfort related to the use of the portable vibration device associated with cryotherapy.

It should be noted that participants who withdraw from the study will be guaranteed the right to be immunized and the health professional will perform the procedure according to the standard protocol of the service and the guidelines of the Ministry of Health. If the participant has contraindications to receive the vaccine in both deltoids, the vaccine may be administered in the ventro gluteal muscle; however, participants who receive the vaccine by this route will not be included in the study.

### Strategies to improve adherence to interventions {11c}

Since this is a one-time intervention, participants will not be required to complete any follow-up time, which facilitates adherence. All participants’ questions about the procedure will be answered at any time during the research. In IG participants, the dwell time of the handheld device on the deltoid prior to vaccine administration will be timed for 30 seconds, so as not to exceed the required time and cause any discomfort that may influence the participants’ acceptance.

### Relevant concomitant care permitted or prohibited during the trial {11d}

Participants having used analgesics in the last six hours prior to vaccination will not be included in the study, in order to avoid influencing the primary outcome—pain.

A companion will be allowed to be present during the administration of the vaccine, if the participant wishes.

### Provisions for post-trial care {30}

The participant will be evaluated in relation to his/her state of anxiety. Thus, if he/she develops any psychological discomfort/shock as a result of the research, he/she will be referred for psychological treatment with a Psychologist Specialist in Cognitive-Behavioral Therapy, at no cost to the participant.

The influenza vaccine, as well as other vaccines, may cause adverse events; if the participant presents any of them, he/she will be referred to a physician specialized in Infectious Diseases and Allergies, at no cost for the participant. In case of severe reactions immediately after the vaccination, the participant will be attended by the health unit staff and, if necessary, the Mobile Emergency Care Service will be requested.

If the participant presents any adverse event at home, he/she must contact the responsible researcher by phone, who will make the referrals and schedule an appointment with the psychologist or doctor of the research team.

In the face of eventual damages, identified and proven, resulting from the research, the participant is assured the right to compensation.

All adverse events will be described, analyzed, and reported.

### Outcomes {12}

#### Primary outcomes

The primary outcome is pain, collected in two instances: before and after vaccination. The primary outcome will be compared between groups (control and intervention), by Student’s *t* test or Mann-Whitney test, and also within groups (before and after vaccine administration) by paired Student’s *t* test or Wilcoxon test, according to the result of normality test.

- Pain: Numerical variable, measured before and after vaccine administration. Data collected from the numerical pain scale, from 0 to 10, being classified as 0 equal to no pain; 1–4 mild pain; 5–6 moderate pain; 7–9 severe pain; and 10 worst pain [23].

#### Secondary outcomes

- Anxiety: Numerical variable, measured before and after vaccine administration, from the visual analog anxiety scale, in which they should mark their anxiety level along a horizontal line with a scale of 0–10 (0 = no anxiety and 10 = highest possible anxiety) [22, 24].
- Satisfaction with vaccine administration: ordinal variable, measured on a 10-point Likert scale (0 = totally dissatisfied; 10 highest possible satisfaction) [22];
- Discomfort caused by the temperature of the frozen bag in contact with the skin: rated only by the IG participants, by a Likert scale (0 = “not at all” to 10 = “extremely uncomfortable” [22];
- Discomfort caused by the vibration of the device in contact with the skin: rated only by the IG participants, by a Likert scale (0 = “not at all” to 10 = “extremely uncomfortable”) [22].

### Participant timeline {13}

The steps of the study will be conducted based on the Consolidated Standards of Reporting Trials (CONSORT) guidelines for reporting clinical trials in a clear, transparent, and comprehensive manner [25]. The protocol is reported according to the Standard Protocol Items: Recommendations for Intervention Trials (SPIRIT) statement [26]. Table 1 presents the operationalization steps of the study.

**Table 1:** Stages of study operationalization 2022

Steps	Study period			
	Enrolment	Allocation	Intervention	Post-intervention
**Timepoint:**	-T_1_	T_0_		T_1_
**Enrolment:**				
Identification of the Study Population - National Influenza Immunization Campaign	X			
Eligibility Criteria	X			
Informed Consent	X			
**Randomisation**				
Allocation of participants		X		
**Interventions**				
Control Group			X	
Intervention Group			X	
**Assessment**				
Sociodemographic Data Collection	X			
Expected level of pain with the vaccine	X			
Self-reported anxiety level before the vaccine	X			
Level of self-reported pain after vaccination	X			X
Self-reported anxiety level after the vaccine				X
Satisfaction with vaccine administration				X
Discomfort caused by the temperature of the frozen bag in contact with the skin				X
Discomfort caused by the vibration of the device in contact with the skin				X

### Sample size {14}

For sample calculation, we used the data available in a previous study that also evaluated the outcomes of pain and anxiety related to administration of Influenza vaccine using or not the Buzzy® device [22]. For the outcome pain, we considered the mean values (± standard deviation) IG = 0.87 (± 0.07) and CG = 1.12 (±0.10). For the anxiety outcome, the values used for the basis of sample calculation were as follows: IG = 1.53 (± 0.13) and CG = 1.48 (± 0.15). The calculation was performed by the software G*power 3.1.9.2, adopting a statistical power of 90% and a significance level of 5%.

For the pain outcome, a sample of 86 participants was estimated in each group, for a total of 172 participants. For the anxiety outcome, a sample of 167 participants was estimated in each group, totaling 334 participants. In order to avoid losses, 350 participants will be selected and randomized to the control and intervention groups.

### Recruitment {15}

The research will take place during the 2022 National Influenza Immunization Campaign, held starting in April. Participants will be recruited in the waiting room while waiting to be vaccinated. An assistant researcher will initially approach the participant and orient them to the research objectives and invite them to participate.

## Assignment of interventions: allocation

### Sequence generation {16a}

The randomization process will be carried out by a researcher external to the study, through the website (http://www.randomization.com/). Seven blocks of 50 people will be generated, with the permutation of the two groups (control group and intervention group). The randomization will be carried out in blocks, because if there is any problem during the study and it is necessary to interrupt it, it will be easier to obtain a balance between the number of participants in each group.

### Concealment mechanism {16b}

After the randomization generation, the same external researcher will distribute the random sequence of participants in each group, in sequentially numbered envelopes, opaque and sealed. The confidentiality of each patient’s allocation will be disclosed to the researchers responsible only at the moment of vaccination administration, when the respective envelopes are opened.

### Implementation {16c}

At the time of vaccine administration, the envelope will be opened by the principal investigator to learn how the IM administration of influenza vaccine will be performed (using or not the portable vibration device in associate with cryotherapy).

## Assignment of interventions: blinding

### Who will be blinded {17a}

The study will be of the open, unblinded type, since it will not be possible for the participant and the researcher to be unaware of the presence or absence of the portable vibration device associated with cryotherapy.

### Procedure for unblinding if needed {17b}

Not applicable, because this study has no blinding step.

## Data collection and management

### Plans for assessment and collection of outcomes {18a}

Before vaccination, an auxiliary researcher will collect data on sociodemographic characterization such as age, gender, and education. Baseline data on the primary outcomes will also be obtained: level of pain expected with the vaccine, from the previously validated Numerical Scale from 0 to 10 [23] and pre-vaccine anxiety level, from the previously validated visual analog anxiety scale, in which they should mark the anxiety level along a horizontal line, containing a scale from 0 to 10 [22, 24].

After vaccination, another assistant researcher will collect the data on pain and anxiety levels after vaccination, for comparison with the values previously collected (baseline) and also for comparison between the groups, control and intervention. The levels of pain and anxiety after vaccination will be obtained from the same instrument used in the pre-vaccination step.

In addition, information will be collected on satisfaction with vaccine administration, measured on a 10-point Likert scale, where 0 = totally dissatisfied and 10 = highest possible satisfaction) [22]. The IG participants will also be asked about the discomfort caused by both the temperature of the frozen bag in contact with the skin and the vibration of the device in contact with the skin, both will be evaluated by a Likert scale in which 0 = “not at all” and 10 = “extremely uncomfortable” [22].

Auxiliary researchers will participate in a training session to perform data collection, based on realistic simulation.

### Plans to promote participant retention and complete follow-up {18b}

Because this is a one-time intervention, there will be no follow-up period, which reduces the possibility of discontinuity. The outcomes will be measured in two moments: before and immediately after vaccination. Participants who drop out of the study and do not submit responses about pain and anxiety levels before and after vaccination will not be included in the study.

### Data management {19}

The data generated by the study will be collected on paper forms and stored for a period of 5 years. The data will be entered independently into spreadsheet software by two auxiliary researchers. The database will be checked by the principal investigator and then exported to the *Statistical Package for Social Science* (SPSS) version 22.0 software. The clinical trial data will be managed and analyzed by one of the researchers who will not participate in the recruitment and intervention to ensure the authenticity, integrity, and privacy of the data during the research process.

### Confidentiality {27}

The researchers will treat the identity of the participants with professional standards of secrecy and confidentiality, following the principles of the Declaration of Helsinki. Participants will be identified only by the initials of their name and coded by the letter P followed by a number, according to the order of allocation (P1, P2, P3...). All information about the participants is confidential and will be used only for the purpose of the research.

### Plans for collection, laboratory evaluation, and storage of biological specimens for genetic or molecular analysis in this trial/future use {33}

Not applicable, as the study does not include biological samples.

## Statistical methods

### Statistical methods for primary and secondary outcomes {20a}

Data will be analyzed using SPSS software, version 22.0. Descriptive and inferential analysis will be performed. The descriptive analysis will use absolute and relative frequency distributions, measures of central tendency (mean and median), and measures of variability (standard deviation and quartiles). The Kolmogorov-Smirnov test will be used to test normality of distribution*.* The primary outcomes (pain) will be compared between groups (control and intervention), by Student’s *t* test or Mann-Whitney test, and also within groups (before and after vaccine administration) by paired Student’s *t* test or Wilcoxon test, according to the result of normality test. The significance level will be 95%. The secondary outcomes, such as anxiety, satisfaction with vaccine administration; discomfort caused by the temperature of the frozen bag in contact with the skin, and discomfort caused by the vibration of the device in contact with the skin, will be used as co-variables in order to identify association with pain. Association of two disabilities with sociodemographic characteristics will also be verified.

### Interim analyses {21b}

The study is considered low risk and all interventions are standardized actions of the vaccination room health team. The data collected will be included in the database within 48 h of the participant’s inclusion in the study; however, the analysis will be performed after all participants are included, given the sample size previously calculated. If there are any adverse events, they will be notified and analyzed immediately by the research team, which will make the decision to terminate the study, if applicable.

### Methods for additional analyses (e.g., subgroup analyses) {20b}

The outcome will be compared between the groups devoted to vaccination: elderly and adult health professionals, in order to verify if there is a difference between pain among them.

### Methods in analysis to handle protocol non-adherence and any statistical methods to handle missing data {20c}

Data from participants who withdraw from the survey and do not submit responses on pain levels before and after vaccine administration will not be analyzed.

### Plans to give access to the full protocol, participant level-data, and statistical code {31c}

Sharing of data, including the full protocol, participant dataset, and statistical codes, will be considered upon reasonable request.

## Oversight and monitoring

### Composition of the coordinating center and trial steering committee {5d}

The trial will be coordinated by professors from the Department of Medicine and Nursing of the Federal University of Viçosa, Brazil. The execution team will be composed of nurses and nursing students from that institution. The data management team will be composed of professors with experience in clinical research and data analysis. The group will meet weekly to evaluate the applicability of the protocol in the pre-intervention period, and daily during the data collection period.

### Composition of the data monitoring committee, its role and reporting structure {21a}

The study has a Data Monitoring Committee (DMC), consisting of 5 members, two nurses, two physicians, and one statistician. The DMC has no conflict of interest in the trial and will be responsible for blinded monitoring and analysis of the data independently and will provide confidential risk reporting to the human research ethics committee in case of suspected unexpected serious adverse events. No interim analysis is planned for this study. The DMC will conduct analysis of the study data on a weekly basis and will advise the Human Research Ethics Committee on continuation, modification, or discontinuation of the study.

### Adverse event reporting and harms {22}

The participant will be instructed to contact the principal investigator to spontaneously report the occurrence of adverse events, which may be local or systemic clinical manifestations. Adverse events will be reported to the National Vaccination System of the Brasilian Ministry of Health.

In addition, auxiliary researchers will conduct telephone contact with participants 24 h and 48 h after vaccine administration to identify the occurrence of any adverse events.

Participants who need medical or other professional attention will be referred, free of charge, by the research team.

### Frequency and plans for auditing trial conduct {23}

There are no scheduled audits, but the Ethics Committee on Human Research of the proposing institution can schedule audits at any time.

### Plans for communicating important protocol amendments to relevant parties (e.g., trial participants, ethical committees) {25}

If it is necessary to make changes in the protocol, these will be forwarded to the Ethics Committee on Human Research of the proponent institution and to the Brazilian Registry of Clinical Trials. After approval by the respective units, they will be communicated to the study participants, who may withdraw their consent at any time.

### Dissemination plans {31a}

The results obtained will be published in peer-reviewed open access journals and presented at national and international conferences, in order to allow wide dissemination. In addition, we intend to produce a video on the technique of administration of influenza vaccine using the portable device of high-frequency vibration associated with cryotherapy and make available the link for free access through the YouTube® channel.

## Discussion

Vaccines are one of the most viable strategies for prevention and control of immunopreventable diseases, among them influenza pneumonia [27]. Despite the importance of mass vaccination adherence, the WHO issued a document warning about the risks of death and illness from immunopreventable diseases due to low vaccination coverage in different countries. The hesitation, delay, or refusal of available vaccine was considered by WHO as one of the ten health threats it intends to combat between 2019 and 2023 [28].

The causes for people’s hesitation are complex and diverse issues, as they involve social, cultural, and economic aspects and can vary from one place to another [11]. Among the main causes are misinformation coupled with strong and persuasive anti-vaccination movements [12, 13]. Although not a new phenomenon, hesitation or refusal of vaccination in the world and in Brazil has been increasing as immunopreventable diseases have disappeared [29]. The Internet in recent years has contributed to the increase of this phenomenon, through social networks with the aim of misinformation of the population, the so-called fake news [30, 31].

Other important factors for non-vaccination or hesitation are physical, geographical and financial availability, safety and confidence in the vaccine, perception of efficacy [32], fear of needles [21], and anxiety [33].

The Influenza vaccine consists of inactivated, fractionated, and purified viruses, characteristics that ensure that it does not cause the disease [10]. However, inactivated vaccines require a greater amount of antigens than attenuated vaccines, in addition to the addition of adjuvants to improve the immune response and increase the protection time, and may cause more pain during administration [34]. The vaccine should be administered IM or subcutaneously [35]. Routinely, the route chosen by professionals is IM; however, it is more painful, because it uses a needle with larger diameter and caliber, capable of penetrating the muscle. In adults, the deltoid muscle is usually selected to receive the vaccine, because at this age, the muscle pain generated after vaccination could temporarily affect ambulation if it was administered in the anterolateral thigh muscle [36].

Although vaccines are safe and effective, they can cause fear, anxiety, and acute pain during IM administration. Although pain is the most common adverse event at this time, and anxiety can worsen it, preventive measures are not routinely used by nursing professionals, which may impact the community’s acceptance of this preventive intervention [37].

Throughout the assistance offered, the nursing team must always be attentive to diagnose the needs of each individual and direct their actions to meet them. It is perceived that acute pain and anxiety resulting from procedures that use needles, such as vaccination, are important nursing diagnoses, which are still neglected by health professionals [38].

In 2015, the WHO recommended some evidence-based interventions for pain management during vaccination, most of which are non-pharmacological interventions, highlighting the use of a portable, disposable device that combines high-frequency vibration associated with cryotherapy [16]. The indicated device is based on the pain gate control theory. It uses the non-painful stimuli of high-frequency vibration and cold applied to the muscle, before and during the generation of the painful stimulus resulting from the administration of the vaccine, thus inhibiting its transmission [19].

Thus, considering the importance of pain for vaccination adherence and the possibility of promoting a better experience with vaccination through low-complexity interventions, this study aims to evaluate the effect of high-frequency vibration associated with cryotherapy during IM administration of influenza vaccine in adults on self-reported levels of pain and anxiety.

The results of this study should be interpreted with caution, since it has limitations such as the absence of blinding of researchers and participants, because during vaccine administration, it will not be possible to mask the intervention, i.e., the use or not of the vibration device and cryotherapy. In addition, it will be performed in a single health care institution. However, it is noteworthy that the unit chosen serves the entire population of the city and a previously calculated sample will be used, based on previous studies, allowing greater representativeness.

It is believed that the results will provide evidence to reorient nursing practice, in search of promoting greater safety and adherence to vaccination campaigns by the population, thus minimizing the occurrence of problems resulting from non-vaccination. Thus, it is expected that the use of non-pharmacological nursing intervention during IM vaccine administration can reduce pain and anxiety in adults, contributing to greater adherence to vaccination, strengthening the National Immunization Program and protecting the individual, family, and community from immunopreventable diseases.

### Trial status

Protocol version 1.0 dated March 17, 2022. The first participant will be recruited when the national Influenza campaign in Brazil begins, scheduled for April 04, 2022. Expected completion June 03, 2022.

## Supplementary Information

**Additional file 1.** Free and informed consent form.

## Data Availability

The final data set will be accessed only by the researchers on the team. Data used or analyzed during the current study will be made available by the corresponding author upon completion of the study analysis and upon reasonable request.

## Abbreviations

WHO: World Health Organization
RCT: Randomized clinical trial
IM: Intramuscular
IG: Intervention group
CG: Control group
CONSORT: Consolidated Standards of Reporting Trials
SPIRIT: Standard Protocol Items: Recommendations for Intervention Trials
SPSS: Statistical Package for Social Science
DMC: Data Monitoring Committee
