# Supplementary material for: Effect of vibration associated with cryotherapy on vaccine-related pain and anxiety levels in adults: study protocol for a randomized clinical trial
Source: Trials. 2022 Aug 1;23:620. doi: 10.1186/s13063-022-06564-7 (PMC9344641; doi:10.1186/s13063-022-06564-7)
Supplement: Supplementary file 1 — Additional file 1. Free and informed consent form. [file 13063_2022_6564_MOESM1_ESM.docx]

**FREE AND INFORMED CONSENT FORM**

You are being invited as a volunteer to participate in the research project "**Effect of a non-pharmacological intervention on pain and anxiety related to the administration of influenza vaccine in adults**. In this research we intend to "**evaluate the effect of a non-pharmacological nursing intervention that uses high-frequency vibration associated with cryotherapy (ice pack) of a device called Buzzy^®^ on the level of self-reported pain and anxiety related to the administration of influenza vaccine by the intramuscular route (IM) in adults.** The reason that leads us to study the management of pain and anxiety levels related to the administration of influenza vaccine intramuscularly in adults is because this procedure is a daily practice of primary care nursing staff. Although pain is the most common adverse event at this moment and anxiety can worsen it, preventive measures are not routinely used by nursing professionals. Evidence-based planning of nursing care in intramuscular vaccine administration will provide comprehensive and individualized care, enabling the implementation of interventions that meet the real needs of each person, minimizing the risks arising from its implementation. Thus, in view of these considerations and the scarce knowledge about the best nursing care during intramuscular administration of influenza vaccine in adults, this research is justified.

The following procedures will be adopted for this study: By agreeing to participate in the study, during intramuscular administration of the influenza vaccine, you may randomly be included in the control group or the intervention group.

If you are included in the control group, you will receive the vaccine in the traditional way as it is already performed annually.

If you are included in the intervention group, you will receive the vaccine in the traditional way as it is already performed annually, but you will have the aid of a high-frequency vibration device associated with cryotherapy (ice pack) placed on your arm 30 seconds before receiving the painful stimulus of vaccination and during the vaccination process. This portable, non-invasive, reusable plastic device called Buzzy^®^ aims to inhibit the transmission of painful stimuli caused by the administration of the vaccine. It has a button to trigger high-frequency vibration and a separate reusable ice pack that is placed on the inside of the device that is in contact with the arm.

Before vaccine administration, the level of pain expected with vaccine administration will be assessed, using a 0 to 10 point scale, where 0 equals no pain; 1-4 mild pain; 5-6 moderate pain; 7-9 severe pain; 10 worst pain. Similarly, the level of anxiety before vaccination will be assessed, from a scale of 0 to 10 points, where 0 corresponds to no anxiety and 10 to the maximum level of anxiety. Immediately after vaccination will be assessed their level of pain felt at the time of vaccination, using and a scale of 0 to 10 points, where 0 equals no pain; 1-4 mild pain; 5-6 moderate pain; 7-9 severe pain; 10 worst pain. Their level of anxiety after vaccination will be assessed, using a scale of 0 to 10 points, where 0 corresponds to no anxiety and 10 to the maximum level of anxiety. Satisfaction with the administration of influenza vaccine by the IM route will be assessed, using a scale of 0 to 10 points, with 0 being totally dissatisfied and 10 being the highest possible satisfaction. For the participants of intervention group (GI), the level of discomfort caused by the temperature of the ice pack of the portable device in contact with the skin and the level of discomfort caused by the vibration of the portable device in contact with the skin will be assessed, using a scale of 0 to 10 points for each variable, where 0 corresponds to not at all and 10 to extremely uncomfortable. All these variables are verified in a simple way, without the need for invasive procedures.

**Benefits:** the benefits of this study include the opportunity to contribute to provide theoretical improvements in the implementation of safe nursing practices during pain and anxiety management during IM vaccination administration, minimizing undesirable effects, contributing to greater adherence to vaccination, strengthening the National Immunization Program (NIP), protecting the individual, family and community from immunopreventable diseases.

**Risks**: the risks arising from participation in this research are grouped into psychological and physical risks. Psychological risks include those related to the possibility of embarrassment and/or discomfort when answering the questions of the data collection instrument, especially the anxiety scale, which assesses a component of mental health: anxiety. Thus, to avoid any diagnostic inference and cause discomfort/psychic trigger, the anxiety scale will be filled only with an X in the number reported by the participant and it will not be possible to identify any score relation, because it will only be calculated at the time of data tabulation by the researchers. You will be placed in a reserved place to answer the questions. In addition, you should be aware that it will be possible to clarify any doubts about the questions to be addressed and that it will be possible to stop filling out the instrument if you feel embarrassed and/or uncomfortable, and that you may withdraw your consent at any stage of the study, having the freedom not to participate in it. Furthermore, if any unforeseen damage or breach of secrecy and confidentiality of the data occurs, compensation will be guaranteed in relation to them. In case of difficulties, you may contact the researcher by e-mail or telephone for further clarification. If you develop any discomfort/psychological trigger, you should contact the responsible researcher, who will immediately provide the necessary psychological care with Psychologist Edinea Batista Freire Dhingra, CRP:04/49857, Specialist in Cognitive Behavioral Therapy.

Regarding physical risks, they include risks arising from the vaccination procedure itself, but that will be minimized by using the appropriate technique and according to the scientific literature. Transitory discomfort may occur due to the contact of the ice pack with the arm. If you have any major discomfort during this administration, you may notify the researcher to stop the evaluation immediately.

Due to the pain and anxiety inherent in IM vaccine administration, you may feel uneasy, dizzy or even faint. To minimize these events, the temperature of the administration room will remain at 21ºC. To reduce the risk of accidents such as falling due to these symptoms, you will be seated in a chair or lying on a stretcher. You will be asked if you have felt ill at other times and will be instructed to report if you begin to feel any of these symptoms so that further care can be implemented immediately.

The Basic Health Unit where the study will take place has qualified physicians, with a minimum residency title in Internal Medicine, who can be called to evaluate the participant and take the necessary steps, and if necessary to refer the participant to a hospital, the service has an ambulance for patient removal.

Influenza vaccine, like other vaccines, can cause adverse post-vaccination events, the vast majority of which are local or systemic clinical manifestations classified as non-severe. The main local symptoms are pain, redness and swelling. To minimize them, do not massage the area after vaccination, in cases of more intense reactions you should apply cold compresses in the first 24 to 48 hours after vaccination, if necessary, use an analgesic prescribed by the healthcare professional. The main systemic manifestations are fever, malaise and body pain. In order to reduce them, rest at home in a well-ventilated place, drink liquids for oral rehydration and, if necessary, use painkillers prescribed by a healthcare professional. If the symptoms do not cease, you should contact the responsible researcher, who will immediately provide the necessary medical care with the physician Alex Pinheiro Simiqueli de Faria, CRM MG 40729, Specialist in Infectology and Allergy.

It is important that you inform the researcher about any local clinical manifestation or assistance to report adverse events following immunization. In addition, you will be contacted by telephone 24 hours and 48 hours after the administration of the vaccine to identify the occurrence of any adverse event.

Another risk involved is infection, which can be minimized with the use of disposable materials, as well as cleaning of the environment and use of aseptic technique throughout the procedure.

At any time during the study, you may notify the researcher to discontinue the evaluation immediately.

It is also emphasized that refusal to participate in the study will not have any future implications. You will receive the vaccine in the same way and will be attended by the staff with the same cordiality.

Participating in this study will not have any cost, nor will receive any financial advantage. However, in the event of any damages, identified and proven, resulting from the research, you have the right to compensation. You are guaranteed complete freedom to refuse to participate or to withdraw your consent at any stage of the research, with no need for prior notice. Your participation is voluntary and refusal to participate will not result in any penalty or change in the way you are cared for by the researcher and the health team of this unit. The results of the research will be available to you when completed. You will not be identified in any publication that may result from this study. Your name or material indicating your participation will not be released without your permission.

This consent form is printed in two original copies, one of which will be filed by the responsible researcher at the Department of Medicine and Nursing at the address: Av. Peter Henry Rolfs, s/n Campus Universitário, Viçosa – MG, Brazil - Zip Code: 36570-900.

The data and instruments used in the research will be stored with the researcher responsible for a period of five years after the end of the research. After this period, they will be destroyed.

The researchers will treat your identity with professional standards of secrecy and confidentiality, in accordance to Brazilian legislation (Resolution 466/2012 of the National Health Council), and will use the information only for academic and scientific purposes.

I,_______________________________________________________contact ______________________________,

I have been informed of the objectives of phase II of the study "**Effect of a non-pharmacological intervention on pain and anxiety related to the administration of influenza vaccine in adults" in a** clear and detailed manner and have clarified my doubts. I know that at any time I may request new information and change my decision to participate if I so wish. I agree to participate. I have received an original copy of this informed consent form and have been given the opportunity to read and clarify my questions.

Luciene Muniz Braga Daskaleas

Av. Peter Henry Rolfs, s/n Campus Universitário (Edifício da Saúde- DEM), Viçosa – MG, Brazil - Zip Code: 36570-900.

Phone: (31) 986513362 E-mail: [luciene.muniz@ufv.br](mailto:luciene.muniz@ufv.br)

In case of disagreement or irregularities under the ethical aspect of this research, you may contact:

CEP/UFV - Ethics Committee on Research with Human Beings

Federal University of Viçosa

Edifício Arthur Bernardes, piso inferior.

Av. Peter Henry Rolfs, s/n - Campus Universitário Cep: 36570-900, Viçosa – MG, Brazil- Zip Code: 36570-900.

Phone: (31)3612-2316 E-mail: [cep@ufv.br](mailto:cep@ufv.br) - www.cep.ufv.br

Viçosa, ______ de ________________ de 20______.

_____________________________________________________

Signature of Participant


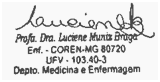


____________________________________________________

Signature of Researcher
